# Corticotropin-releasing hormone deficiency results in impaired analgesic response during CFA-induced inflammation

**DOI:** 10.1007/s42000-024-00565-8

**Published:** 2024-05-14

**Authors:** Efthymia Karagianni, Olga Rassouli, Smaragda Poulaki, Eirini Dermitzaki, George Liapakis, Andrew N. Margioris, Maria Venihaki

**Affiliations:** 1 Department of Clinical Chemistry, School of Medicine, University of Crete, Heraklion, Greece; 2 Department of Pharmacology, School of Medicine, University of Crete, Heraklion, Greece

**Keywords:** CRH, Glucocorticoid, Pain, Inflammation, Cytokine

## Abstract

**Purpose:**

Corticotropin-releasing hormone (CRH) plays an important role in relief of pain by releasing analgesia-associated molecules in several inflammatory states. During inflammation, peripheral CRH acts on cells of the immune system to stimulate the local expression of proopiomelanocortin (POMC) and the production of β-endorphin, which in turn binds to opioid receptors on sensory neurons to produce antinociception. In the present study, we further investigated the role of endogenous CRH in inflammatory pain by determining the effects of *Crh*-deficiency on this process.

**Methods:**

For this purpose, we used *Crh*-deficient (*Crh-/-)* mice and their wildtype (*Crh* + */* +*)* littermates in the CFA (Complete Freund’s Adjuvant)-induced inflammatory pain model. Pain thresholds were evaluated with the Hargreaves apparatus.

**Results:**

Our experiments showed that *Crh* deficiency led to increased pain response, which was associated with decreased POMC mRNA levels in locally inflamed paws of these mice. Furthermore, *Crh-/-* mice had higher paw edema than *Crh* + */* + mice. Histological evaluation of inflamed paw tissues revealed increased inflammatory response in *Crh-/-* mice. Protein levels of proinflammatory cytokines, such as IL-6, TNF-α, and IL-1β, were higher in inflamed tissue of *Crh-/-* mice compared to wildtype mice. Corticosterone replacement increased the pain threshold of *Crh-/-* mice, restored their paw volume to the levels of wildtype mice, and significantly reduced their proinflammatory cytokine levels. Furthermore, glucocorticoid administration significantly increased POMC mRNA expression in the inflamed paw.

**Conclusion:**

Our data suggest that genetic deficiency of CRH is associated with increased pain. This effect is likely attributable to the accompanying glucocorticoid insufficiency and is in part mediated by opioids expressed locally.

## Introduction

Inflammatory diseases, such as rheumatoid arthritis, are characterized by an intense sensation of pain. Until now, the mechanisms involved in pain response were attributed solely to actions of the nervous system. However, several studies have revealed that factors producing analgesia are expressed in peripheral tissues as well [1, 2]. Indeed, it has been shown that endogenous opioid peptides are released by immune cells which are recruited in the inflamed tissue and which include β-endorphin (END), met-enkephalin (ENK), and dynorphin (DYN). The type of immune cells that mediate this effect depends on the particular stage of the inflammatory response [3–6]. Furthermore, under inflammatory and other pathological conditions, various types of immune cells contain and produce corticotropin-releasing hormone (CRH). Peripheral “immune” CRH has potent proinflammatory properties and its absence results in decreased inflammatory response in certain models of inflammation in contrast to its anti-inflammatory effects through the release of adrenal glucocorticoid [7–9]. Both the proinflammatory and anti-inflammatory effects of CRH are mediated by the CRF receptors, CRFR1 and CRFR2 [10, 11].

In addition to its dual role in immune cells, peripheral CRH can induce the expression of proopiomelanocortin (POMC), the precursor molecule of β-endorphin [12] in immune cells and thus can elicit analgesia [11, 13]. Indeed, it has been shown that local administration of CRF into inflamed tissue causes pain inhibition in rats [14–16]. The analgesic effect of CRH is also supported by clinical data in patients with acute knee trauma [17]. Most of these experiments, however, that have investigated the role of CRH in antinociception involved mainly pharmacological studies or studies in which the synthesis and/or the action of the peptide was abolished locally [13]. The aim of our study was to clarify the consequences of the complete absence of endogenous CRH, and thus of the resulting insufficiency of glucocorticoid, in inflammatory pain response and to thereby seek to clarify the role of CRH. For this purpose, we used the *Crh-/-* deficient mice [18] treated with or without exogenous glucocorticoid in the CFA-induced inflammation mouse model.

## Material and methods

### Animal housing

*Crh-/-* mice and their wildtype littermates (*Crh* + */* +) of 129SVxC57BL/6 genetic background (generated as we have previously described [19]) were kept in a 12:12 h light/dark cycle, with water and food provided ad libitum. All experiments herein presented were performed in male mice of 2–4 months of age, unless otherwise stated (3–5 animals per experiment/per genotype/per condition). The animals were housed individually at least 24 h before each experiment. All experiments and animal care had been approved by the Committee of Experimental Animal Protocols of the University of Crete and the Veterinary Department of Crete, and were in accordance with the International Association for the Study of Pain.

### Induction of inflammation

Mice of both genotypes were injected with 20 μl CFA (Complete Freund’s Adjuvant) (F5881, Sigma, Germany) into their left hind paw under mild anesthesia. The contralateral hind paw remained untreated and served as control. All injections and experiments were performed on the morning of the experimental day 1 h after the lights had been turned on.

### Algesiometry testing

Pain response was measured using the Hargreaves Plantar Test Apparatus (Ugo Basile, Italy) [20]. *Crh-/-* and *Crh* + */* + mice were placed into a clear plastic chamber on top of a glass floor and were left in this compartment for at least 20 min every day for a total period of 5 days (acclimation period). On the day of the experiment, the mice were injected with 20 μl CFA, as previously described [21]. Before the measurements, the mice were left 15–20 min in their chambers to adapt properly. A movable IR source was positioned under the glass panel directly beneath the hind paw and measurements of paw withdrawal latencies (the time that the animal takes to withdraw the paw from the thermal stimulus) were taken according to the manufacturer’s instructions. Pilot experiments were performed to set the intensity of the IR (IR 30). The mean of three measurements was taken at each time point (3, 6, and 24 h post CFA injection) for both hind paws.

### Paw edema evaluation

The inflammation-associated edema was measured using a plethysmometer at the same time points that pain response was measured (3, 6, and 24 h post CFA injection). The inflamed paw was immersed in the plethysmometer buffer (0.08% NaCl and 0.2% wetting compound) and the volume of the buffer that was displaced was proportional to the volume of the edema.

### Corticosterone replacement

*Crh-/-* mice received either corticosterone (Sigma, Germany) at a final concentration of 10 μg/ml or vehicle (ethanol) in their drinking water for a total period of 4 days before the induction of inflammation.

### Evaluation of plasma corticosterone levels

Blood was collected via the retro-orbital route. Samples were centrifuged at 3000 rpm for 10 min at 4° C. Plasma was isolated and stored at -80° C until further use. Plasma corticosterone levels were measured with a commercial RIA kit (MP Biomedicals, USA).

### Measurement of cytokine production

Inflamed paw tissues were homogenized in 1 × PBS (Phosphate Buffer Saline, pH 7, GIBCO, Life Technologies, UK) with 1 × Protease Inhibitors (Roche, Germany) final concentration. IL-6, IL-1beta (IL-1β), and TNF-alpha (TNF-α) protein levels were evaluated in tissue lysates using commercial ELISA kits (Biolegend, Inc, San Diego, USA) according to the manufacturer’s instructions. A small quantity of each sample was kept for total protein evaluation using the Bradford assay (Coomassie Blue, Biorad, Germany). The same ELISA kits were used to evaluate IL-6 and TNFα protein levels in plasma of CFA-treated animals.

### RNA isolation and cDNA synthesis

Inflamed paw tissues were homogenized in TRI Reagent (Sigma, Germany). RNA isolation was performed as previously described [21]. The efficiency and the quality of the RNA extraction method were analyzed in a NanoDrop 2000 UV–Vis Spectrophotometer (Thermo Scientific). The RNA samples were then immediately processed to synthesize cDNA so as to minimize possible breakdown due to repeating freeze–thaw cycles. The PrimeScript First Strand cDNA Synthesis kit (Takara, Bio, Inc., Japan) was used to synthesize cDNA from total extracted RNA in the presence of random hexamer primers.

### Semi-quantitative and real time PCR

The primers for the specific mRNAs were obtained from VBC-Biotech Service GmbH (Vienna, Austria). The sequences of the sense and antisense primers used, the annealing temperature, the cycles, and the product size are listed in Table 1. A total of 1-2 μl of cDNA was amplified for each reaction. For semi-quantitative PCR, KK1015 PCR kit (KapaBiosystems, MA, USA) was used, except for the δ- opioid receptor where KK5701 HotStart ReadyMix (KapaBiosystems, MA, USA) was used. The amplification reactions were analyzed using 1.5–2% agarose gels. For real time PCR, reaction amplification was performed in an ABI PRISM 7000 Real-Time PCR apparatus (AB Applied Biosystems StepOnePlus Real Time PCR System for UCN3) for a maximum of 45 cycles using the KAPA SYBR® FAST qPCR Master Mix (KapaBiosystems, MA, USA). β-actin was used as the reference gene in all reactions.

**Table 1:** Sequence of RT-PCR primers and products size

Primer	Annealing temperature °C/cycles	Product size (bp)
β-actin sense: 5’-TCAGAAGGACTCCTATGTG-3’ β-actin antisense: 5’-TCTCTTTGATGTCACGCAC-3’	55/25	499 [7]
POMC sense: 5’-CTGCTTCAGACCTCCATAGATGTG-3’ POMC antisense: 5’-CAGCGAGAGGTCGAGTTTGC- 3’	60/45	120 [33]
μ-opioid receptor sense: 5’-ACGCTCAGACGTTCCATTCT-3’ μ-opioid receptor antisense: 5’-CCAAAGAGGCCCACTACAC-3’	59/45	434 [34]
κ-opioid receptor sense: 5’-CAGCTCTTGGTTCCCCAACTG-3’ κ-opioid receptor antisense: 5’-TGCAAGGAGCATTCAATGACATC-3’	59/45	561 [35]
δ-opioid receptor sense: 5’-GTGCAAGGCTGTGCTCTCCATTG-3’ δ-opioid receptor antisense: 5’-GTCGGGTAGGTCAGGCGGCAGCGCCACCG-3’	64/35	770 [34]
CRF sense: 5’-AGCCCTTGAATTTCTTGCA-3’ CRF antisense: 5’-AACACGCGGAAAAAGTTA-3’	60/40	202 [36]
CRFR1 sense: 5’-GCCGCCTACAACTACTTCCA-3’ CRFR1 antisense: 5’-CGGAGTTTGGTCATGAGGAT-3’	60/45	320 [36]
CRFR2 sense: 5’- CTGGTGGCTGCTTTCCTGCTTTTC-3’ CRFR2 antisense: 5’-ATGGGGGCCCTGGTAGAT GTAGTCC-3’	60/45	426 [36]

#### H&E staining

Isolated paw inflamed tissues were immediately fixed in 4% cold paraformaldehyde in PBS and incubated o/n at 4° C. The next day, they were washed with 1 × PBS and transferred in 30% sucrose in PBS solution until the tissue sank to the bottom of the tube. The tissues were finally placed in 100% OCT (Tissue Freezing medium, Leica Biosystems, Germany) and frozen in dry ice. The samples were kept at -80° C until use. Sections (10 μM) were cut in a cryostat (Leica CM 1860 UV, Germany) and were stained with hematoxylin and eosin (H&E) according to standard protocols.

#### Column chromatography and evaluation of β-endorphin peptide levels

Inflamed hind paws were homogenized in 500 μl HCl 0.1N (Sigma Aldrich, Germany). Tissue homogenates were centrifuged at 12,000 × g for 20 min at 4 °C and stored at -80 °C until use. For the isolation of intracellular peptides, the columns (SEP- pack C18, Water associated, ΜΑ, USA) were washed with 10 ml acetonitrile (Lab-Scan Analytical Sciences) and 20 ml HCl 0,1N. Next, the samples were placed in the column and the hydrophobic peptides were retained. The columns were washed again with 20 ml HCl 0.1N to remove the chemical compounds of high polarity. The peptides were eluted with 3 ml of acetonitrile/HCl 0.01N (4:1) solution. The samples were concentrated using a vacuum concentrator centrifuge (Univapo UVC 150 H) and kept at -20° C until use. β-endorphin concentration was measured with an ELISA kit (ΕΚ-022–06, Phoenix Pharmaceuticals Inc, USA), according to the manufacturer ‘s instructions. The results were normalized with the concentration of the total protein of the tissues.

#### Statistics

In all experiments, each group consisted of three to six mice and each individual experiment was performed at least twice. The data were analyzed with the Mann–Whitney and Kruskal–Wallis tests, followed by Dunn’s multiple comparisons test. The results are expressed either as mean ± SEM or median (min, max) or median (25th, 75th percentile). For all analyses, *P* < 0.05 was considered significant. Statistical analysis was performed using IBM SPSS Statistics software and GraphPad Prism software. Quantification of PCR results was performed using Tina Scan software.

## Results

### Evaluation of paw withdrawal latencies

**Fig. 1:**
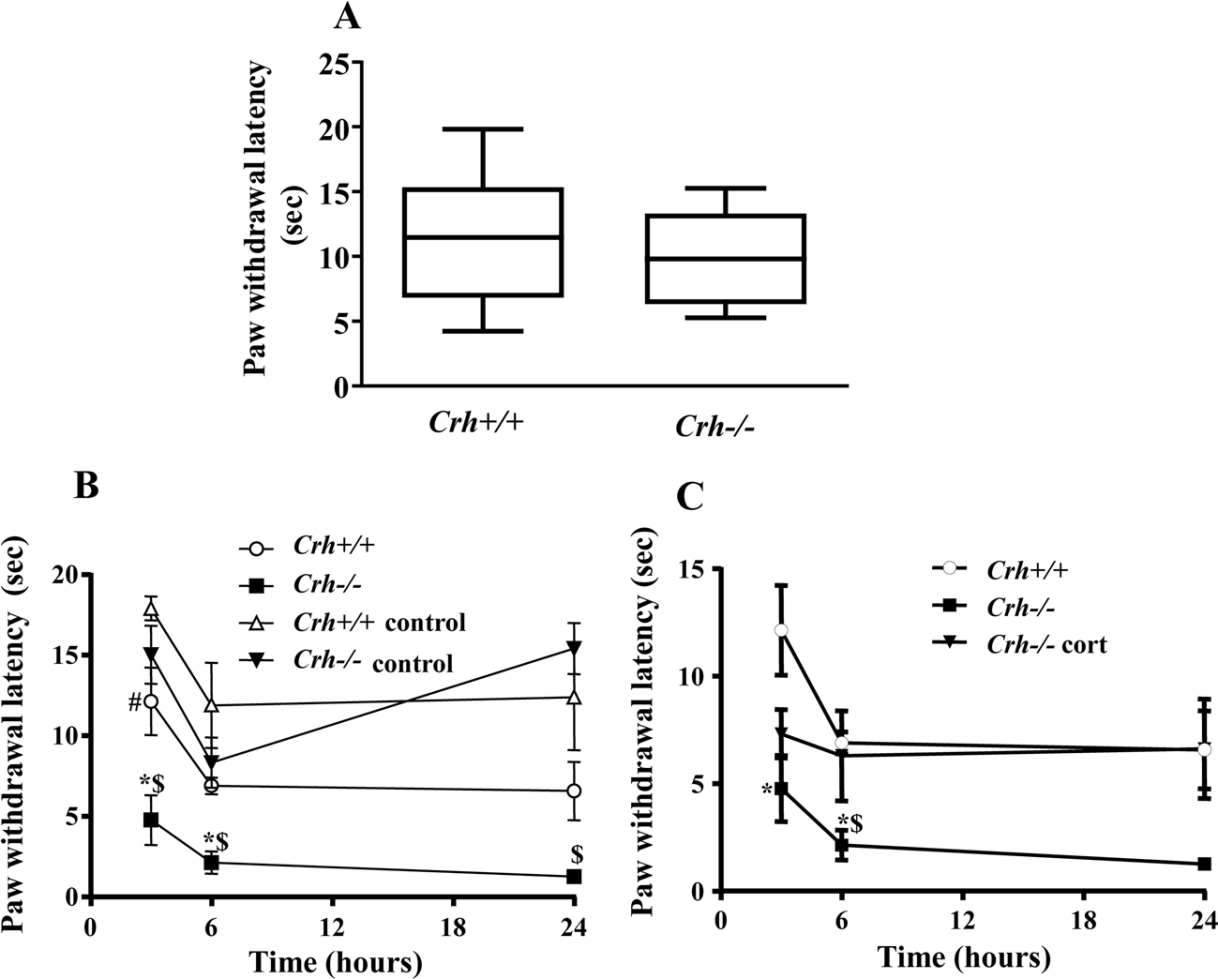
Basal and CFA-induced pain response of *Crh* + */* + and *Crh-/-* mice**. A**. Basal paw withdrawal latencies to a beam of radiant heat were assessed by the Hargreaves Plantar Test Apparatus. For each mouse, the mean of three separated measurements of both hind paws was estimated. Data were analyzed using the Mann–Whitney test and are expressed as median (min to max) (*P* = 0.572. *n* = 4–5 mice/genotype/experiment). **B.** Effect of CFA-induced inflammation on pain response. *Crh* + */* + and *Crh-/-* mice were injected with 20 μl CFA in the left hind paw. The right hind paw served as control. Paw withdrawal latencies were assessed 3-, 6-, and 24-h following CFA injection. Data are expressed as mean ± SEM. (3 h *:*P* = 0.016, between mice of the two genotypes under the same condition, #:*P* = 0.048, between the hind paws of *Crh* + */* + mice exposed to the different treatment, $:*P* = 0.020 between the hind paws of *Crh-/-* mice exposed to the different treatment. *n* = 3–4 mice/genotype/treatment/experiment; 6 h *:*P* = 0.014, between mice of the two genotypes under the same condition, ^$^:*P* = 0.014 between the hind paws of *Crh-/-* mice exposed to the different treatment. *n* = 3–4 mice/genotype/treatment/experiment; 24 h ^$^:*P* = 0.010 between the hind paws of *Crh-/-* mice exposed to the different treatment. *n* = 3–4 mice/genotype/treatment/experiment; The test used was Kruskal–Wallis, followed by Dunn’s multiple comparison test). **C**. Effect of glucocorticoid replacement on pain response. *Crh-/-* mice were treated with corticosterone in their drinking water for 4 days before the induction of inflammation. Data are expressed as mean ± SEM. (3 h *:*P* = 0.027, between mice of the two genotypes under the same condition, *n* = 3–4 mice/genotype/treatment/experiment; 6 h *:*P* = 0.018, between mice of the two genotypes under the same condition, ^$^:*P* = 0.048, between *Crh-/-* mice exposed to different treatment. *n* = 3–4 mice/genotype/treatment/experiment; The test used was Kruskal–Wallis, followed by Dunn’s multiple comparison test)

We first evaluated the basal levels of pain in *Crh* + */* + and *Crh-/-* mice using the Hargreaves test following the 4-day period of acclimation. No differences were found between the two genotypes (Fig. 1A). For the evaluation of the effect of *Crh* deficiency on inflammatory pain, mice from both genotypes were injected with CFA into the left hind paw, while the right paw was used as control (no injection). Measurement of the response of both paws of each mouse to a thermal stimulus showed that CFA-injected paws of both genotypes had shorter withdrawal latencies compared to their untreated contralateral paws (Fig. 1B, signs # and $, Table 2), as expected. The comparison of the pain response of the CFA-treated paws between the two genotypes revealed that *Crh-/-* mice had shorter withdrawal latencies compared to *Crh* + */* + mice 3 and 6 h following the induction of inflammation (Fig. 1B, sign *, *P* < 0.05, Table 2), while no difference was observed between the untreated paws of mice of both genotypes at all time points tested (Fig. 1B, Table 2.

**Table 2:** Data are presented as median [25% percentile, 75% percentile]; (min–max)

	PAW WITHDRAWAL LATENCIES (sec)					
	3 h		6 h		24 h	
Genotype	median [25%, 75%]	**N**	median [25%, 75%]	**N**	median [25%, 75%]	*N*
	(min–max)		(min–max)		(min–max)	
*Crh* + */* +	13.67 [13.33, 15.50]	3	6.75 [5.99, 7.93]	4	3.67 [3.53, 8.07]	3
	(13.33–15.50)		(5.93–8.13)		(3.53–8.07)	
*Crh-/-*	4.18 [2.18, 7.96]	4	1.43 [0.93, 2.07]	3	1.17 [0.97, 1.63]	3
	(2.00–8.73)		(0.93–2.07)		(0.97–1.63)	
*Crh* + */* + control	17.87 [16.49, 19.34]	4	11.25 [7.33, 17.09]	4	13.10 [6.00, 18.06]	4
	(16.27–19.60)		(6.13–18.93)		(3.70–19.65)	
*Crh-/-* control	15.08 [11.51, 18.46]	4	8.75 [5.20, 11.02]	4	14.00 [13.67, 18.57]	3
	(10.97–18.93)		(4.18–11.63)		(13.67–18.57)	

Statistics in paw withdrawal latencies (sec) presented in Fig. 1B

### Evaluation of paw inflammatory response

**Fig. 2:**
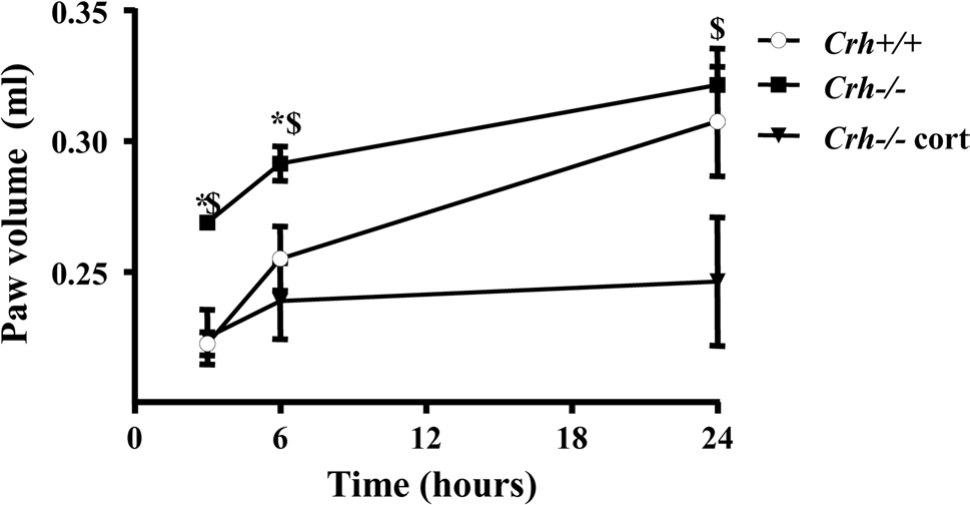
Effect of CFA-induced inflammation on paw edema. *Crh* + */* + and *Crh-/-* mice were injected with 20 μl CFA in the left hind paw. Paw volumes were assessed 3-, 6-, and 24-h following CFA injection. Data are expressed as mean ± SEM and a representative experiment is shown of at least two independent experiments. (3 h *:*P* = 0.028, between mice of the two genotypes under the same condition, ^$^:*P* = 0.028, between *Crh-/-* mice exposed to different treatment; 6 h *:*P* = 0.050, between mice of the two genotypes under the same condition, ^$^:*P* = 0.027, between *Crh-/-* mice exposed to different treatment. *n* = 4 mice/genotype/treatment; 24 h ^$^:*P* = 0.047, between *Crh-/-* mice exposed to different treatment. *n* = 4 mice/genotype/treatment; The test used was Kruskal–Wallis, followed by Dunn’s multiple comparison test)

**Fig. 3:**
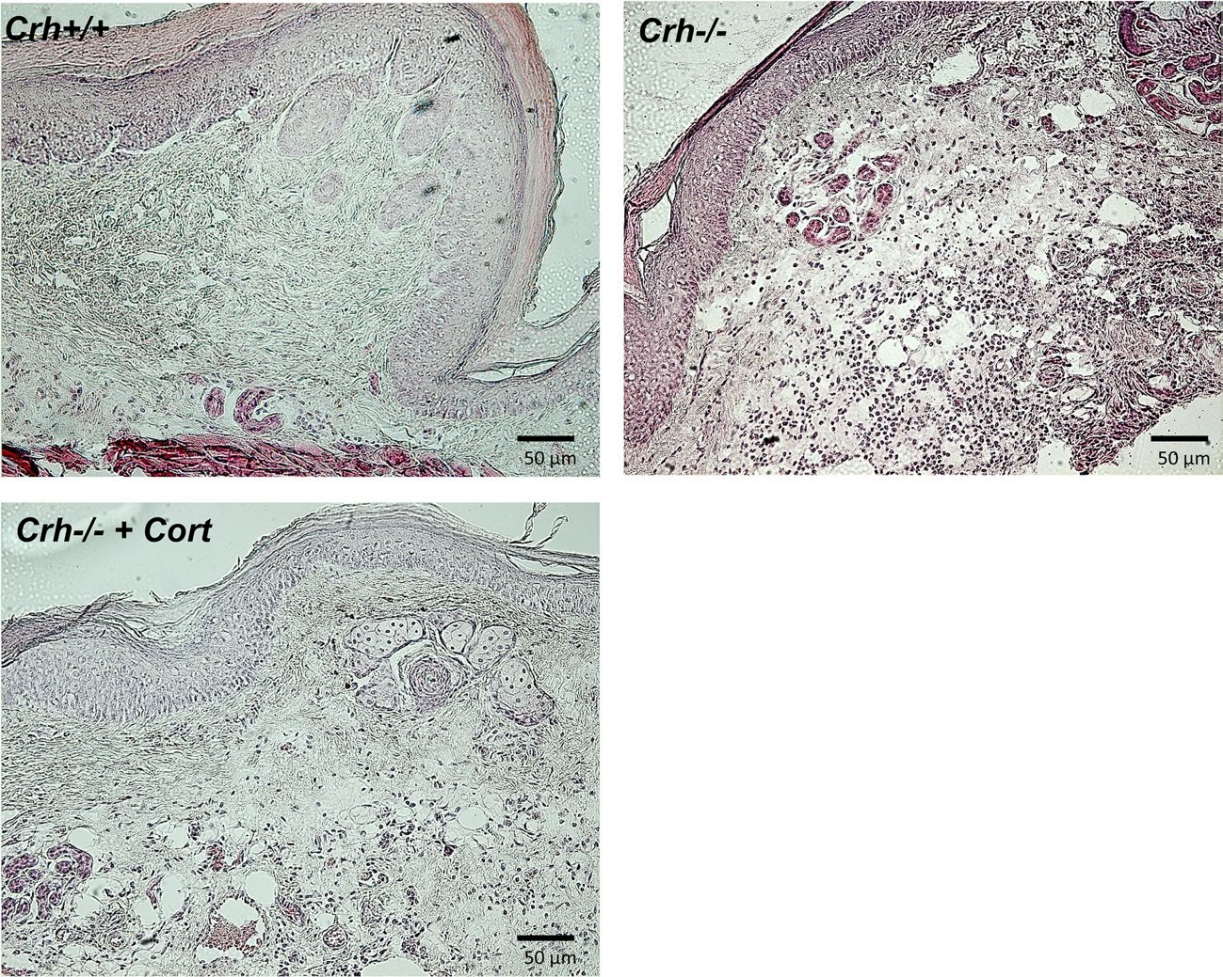
Histological evaluation of inflamed paws**.** Representative sections of CFA treated paws, 6 h after the injection, from (**A**) *Crh* + */* + and (**B**) *Crh–/–* mice stained with H&E. *Crh–/–* mice had higher leukocyte infiltration than *Crh* + */* + . (**C**) Corticosterone replacement in the drinking water of *Crh–/–* mice improved the histological picture. Scale bar was 50 μm

Three hours after the intraplantar injection of CFA, mice of both genotypes developed local (paw) inflammation which was characterized by redness and swelling, as this was estimated by the increased paw volume (Fig. 2, Table 3). The edema was more intense in *Crh-/-* mice compared to wildtype mice. In fact, *Crh-/-* mice showed a higher edema formation 3 h after the induction of inflammation, which remained elevated for up to 24 h (Fig. 2, Table 3). Histological evaluation of the inflamed areas from both genotypes was performed 6 h after the induction of inflammation, a time point at which the mice of both genotypes showed the shorter withdrawal latencies. CFA-injected *Crh* + */* + and *Crh-/-* mice demonstrated a significant inflammatory response (Fig. 3A and B, respectively). The inflammatory response of the *Crh-/-* mice (as evaluated by a blinded investigator) was significantly higher than that of their wildtype littermates, as judged by the leukocytic infiltration of the inflamed area.

**Table 3:** Data are presented as median [25% percentile, 75% percentile]; (min–max)

	PAW VOLUME (ml)					
	3 h		6 h		24 h	
Genotype	median [25%, 75%]	*N*	median [25%, 75%]	*N*	median [25%, 75%]	*N*
	(min–max)		(min–max)		(min–max)	
*Crh* + */* +	0.23 [0.21, 0.23]	4	0.26 [0.23, 0.28]	4	0.31 [0.27, 0.34]	4
	(0.21–0.23)		(0.23–0.28)		(0.26–0.35)	
*Crh-/-*	0.27 [0.27, 0.27]	4	0.29 [0.27, 0.31]	4	0.33 [0.29, 0.35]	4
	(0.27–0.28)		(0.27–0.31)		(0.29–0.35)	
*Crh-/-*	0.23 [0.21, 0.25]	4	0.24 [0.21, 0.26]	4	0.24 [0.21, 0.30]	4
+ Cort	(0.20–0.25)		(0.20–0.27)		(0.21–0.31)	

Statistics in paw volume (ml) presented in Fig. 2

### Cytokine levels in local inflamed paw tissue

**Fig. 4:**
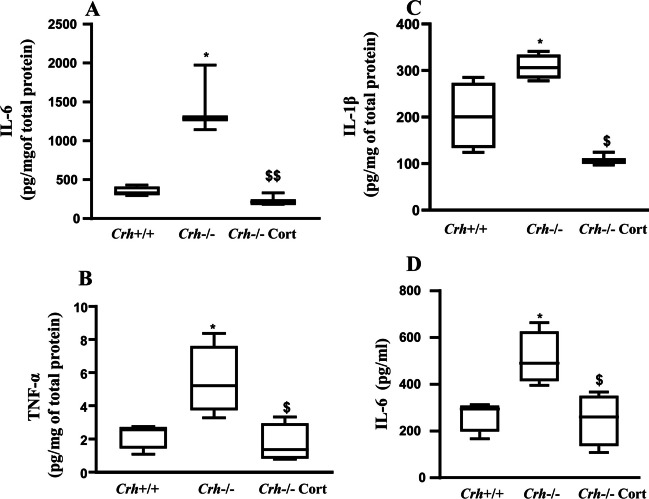
Cytokine levels in inflamed paw tissues and plasma. IL-6 (**A**), TNF-α (**B**), and IL-1β (**C**) levels were evaluated in inflamed paw tissues. Plasma IL-6 (D). Cytokine levels were assessed with ELISA in inflamed paws or plasma collected 6 h following the injection of CFA. Data of all panels are expressed as median (min to max) and a representative experiment is shown of at least two independent experiments. (**A**)*P = 0.035, ^$$^*P* = 0.002 (**B**)*P = 0.028, ^$^*P* = 0.013 (**C**) *P = 0.034, ^$^*P* = 0.019 (**D**) *P = 0.025 and ^$^*P* = 0.021(*: indicates difference between mice of the two genotypes under the same condition, and ^$^: indicates difference between *Crh-/-* mice exposed to different treatment. *n* = 4 mice/genotype/treatment; The tests used were either Mann–Whitney or Kruskal–Wallis, followed by Dunn’s multiple comparison test)

Tissue (inflamed paw) and plasma proinflammatory cytokines (IL-6, TNF-α, and IL-1β) were evaluated 6 h following the injection of CFA. Tissue protein levels of IL-6, TNF-α, and IL-1β were significantly elevated in *Crh-/-* mice compared to their wildtype littermates (Fig. 4A, B, and C, respectively). Plasma IL-6 and TNF-α levels, measured at the same time point, were markedly increased in both *Crh* + */* + and *Crh-/-* mice (Fig. 4D and data not shown, respectively), while plasma IL-1β levels were undetectable. In *Crh-/-* mice, tissue and plasma IL-6 levels were two-to-three-fold higher than those of *Crh* + */* + mice.

### Expression of opioid peptides and the CRF system in inflamed paw tissue

**Fig. 5:**
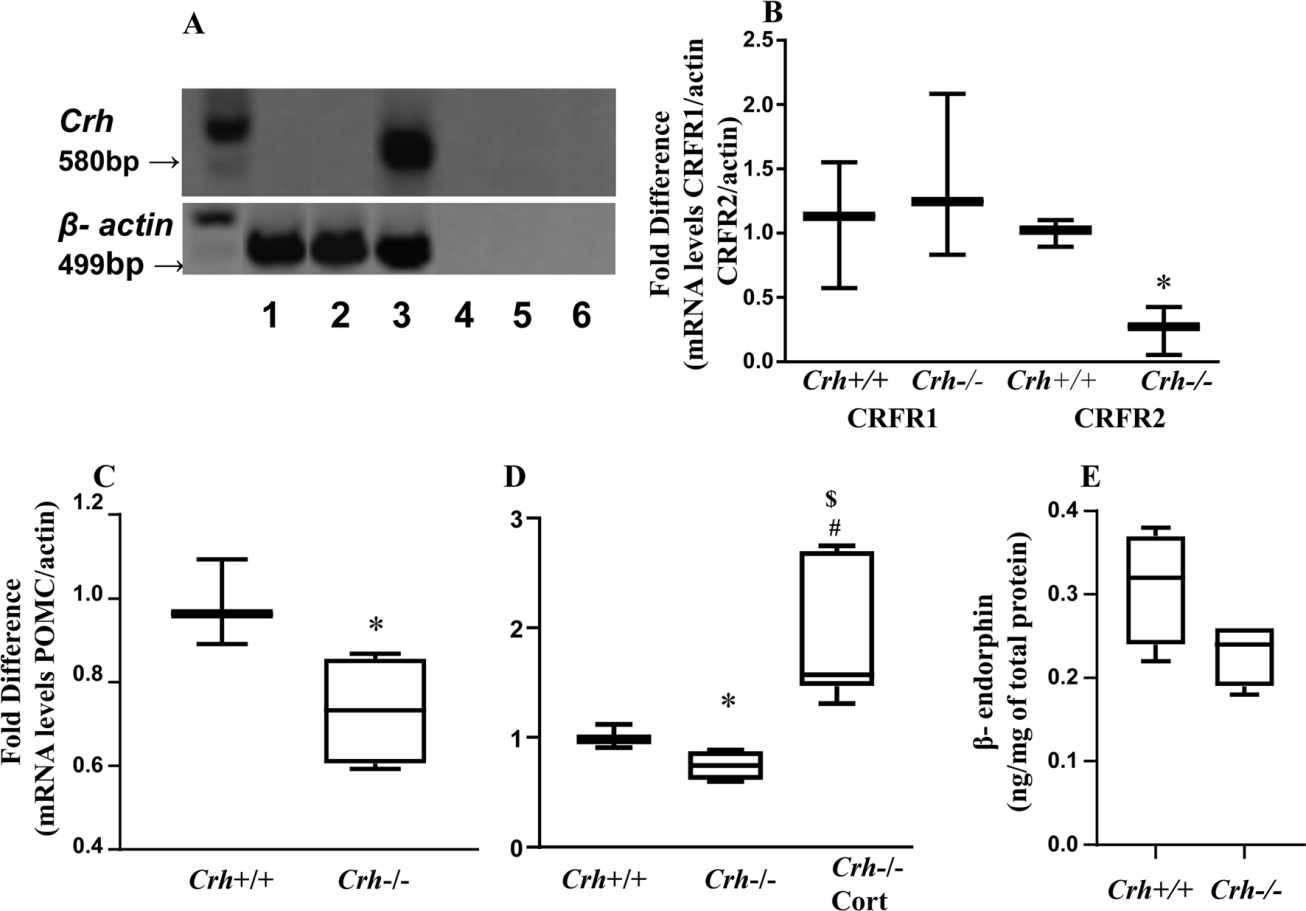
Expression of CRH and its receptors and POMC and β-endorphin in inflamed tissue. CRH mRNA in inflamed paw was assessed with RT-PCR (**A**) 6 h following the injection of CFA (Lane 1, inflamed paw from *Crh* + */* + mice; lane 2, inflamed paw from *Crh-/-* mice; lane 3, brain from *Crh* + */* + mice; lanes 4 and 5, No-RT samples and lane 6, negative control). **B** CRFR1 and CRFR2 mRNA expression in inflamed paw was assessed 6 h following CFA injection (*:*P* = 0.05 between mice of the two genotypes under the same condition *n* = 3 mice/genotype/treatment; The test used was Mann–Whitney for comparison of each receptor mRNA expression between the two genotypes). Both receptors’ mRNA expression was normalized with the corresponding β-actin mRNA expression. POMC mRNA expression in inflamed paw was assessed with Real Time PCR (**C**) 6 h and (**D**) 24 h, following the injection with CFA. (*: *P* = 0.028, between mice of the two genotypes. *n* = 4 mice/genotype/treatment; ^$^:*P* = 0.004 between *Crh-/-* mice exposed to the different treatment. *n* = 4 mice/genotype/treatment/experiment; #:*P* = 0.020 between *Crh* + */* + and *Crh-/-cort* mice. The tests used were either Mann–Whitney or Kruskal–Wallis, followed by Dunn’s multiple comparison test). POMC mRNA expression was normalized with the corresponding β-actin mRNA expression. (**E**) β-endorphin protein levels in inflamed paw, as measured with ELISA, 24 h after CFA injection (*n* = 4 mice/genotype; Mann–Whitney). All data are expressed as median (min to max)

The main factors involved in peripheral pain response include CRH and its receptors as well as opioid peptides and their receptors expressed in the inflamed tissue [3, 10]. Therefore, we investigated the relative expression of these factors in both genotypes. As expected, Crh mRNA was not expressed in inflamed paw tissues isolated from *Crh* deficient mice (Fig. 5A, lane 2) and, to our surprise, no expression was also identified in wildtype mice (Fig. 5A lane 1), while a product of the expected molecular size was detected in brain tissue (positive control) isolated from wildtype mice (Fig. 5A, lane 3). The mRNAs of CRFR1 and CRFR2 were expressed in the inflamed tissues of both genotypes (Fig. 5B). Interestingly, the mRNA levels of CRFR1 did not differ between the two genotypes, while the mRNA levels of CRFR2 were lower in *Crh-/-* mice compared to their wildtype littermates. Inflamed tissue POMC mRNA expression, the precursor of endorphins, was significantly lower in *Crh-/-* mice compared to their *Crh* + */* + littermates 6 and 24 h post CFA injection (Fig. 5C, D). However, no difference was found in β-endorphin protein levels 24 h following CFA injection (Fig. 5E). No difference was observed in P-enkephalin (PENK) mRNA expression between the two genotypes (data not shown). Expression of mu opioid receptor mRNA was at similar levels in the inflamed tissue of both genotypes, whereas kappa and delta opioid receptor mRNAs were undetectable (data not shown) in the inflamed paws.

### Response to CFA following glucocorticoid replacement

To investigate whether the altered pain and inflammatory response of *Crh-/-* mice was due to CRH deficiency or to the associated glucocorticoid insufficiency, we next evaluated these responses (pain and inflammation) as well as cytokine levels in *Crh* + */* + and *Crh-/-* mice that had their corticosterone clamped to the same level by glucocorticoid administration in the drinking water of the latter (*Crh-/-*cort mice) for 4 days before the induction of inflammation. Leukocyte infiltration of *Crh-/-* mice, treated with corticosterone, was similar to that of *Crh* + */* + inflamed mice (Fig. 3C). Similarly, edema did not differ between *Crh* + */* + and *Crh-/-*cort inflamed mice at 3 and 6 h (Fig. 2B). At 24 h, however, paw volume of *Crh-/-*cort mice remained lower compared that of *Crh* + */* + mice (Fig. 2B). Moreover, tissue levels of IL-6, TNF-α, and IL-1β and plasma levels of IL-6 of *Crh-/-* corticosterone-treated mice were significantly reduced compared to untreated *Crh-/-* mice and were comparable to the levels of *Crh* + */* + mice (Fig. 4A, B, C, and D, respectively). Even more interestingly, corticosterone replacement of *Crh-/-* mice significantly increased their pain threshold to that measured in *Crh* + */* + mice (Fig. 1C, Table 4). Similarly, tissue POMC mRNA levels were significantly increased following corticosterone replacement (Fig. 5D).

**Table 4:** Data are presented as median [25% percentile, 75% percentile]; (min–max)

	PAW WITHDRAWAL LATENCIES (sec)					
	3 h		6 h		24 h	
Genotype	median [25%, 75%]	*N*	median [25%, 75%]	*N*	median [25%, 75%]	*N*
	(min–max)		(min–max)		(min–max)	
*Crh* + */* +	13.67 [13.33, 15.50]	3	6.75 [5.99, 7.93]	4	3.67 [3.53, 8.07]	3
	(13.33–15.50)		(5.93–8.13)		(3.53–8.07)	
*Crh-/-*	4.18 [2.18, 7.96]	4	1.43 [0.93, 2.07]	3	1.17 [0.97, 1.63]	3
	(2.00–8.73)		(0.93–2.07)		(0.97–1.63)	
*Crh-/-*	7.03 [5.53, 9.37]	3	7.45 [4.08, 16.82]	4	4.45 [1.39, 9.68]	4
+ Cort	(5.53–9.37)		(3.97–18.93)		(0.83–10.97)	

Statistics in paw withdrawal latencies (sec) presented in Fig. 1C

## Discussion

In the present study, we used the *Crh*-deficient mouse model to clarify the role of endogenous CRH in inflammatory pain and differentiate its local peripheral effect from the central effect arising from the stimulation of the HPA axis and the production of the anti-inflammatory glucocorticoid. In our experiments, we used only male mice to avoid inconsistencies between the two genders. Indeed, most research and preclinical pain studies have been conducted exclusively in male animals given that studies that included females have shown significant sex differences in the physiological mechanisms underlying pain [22]. We found that *Crh* deficiency is associated with decreased pain threshold following the CFA-induced inflammation, an effect which was, however, reversed by the administration of glucocorticoid. We also found that lack of CRH impairs the inflammatory response in the CFA-induced inflammation mouse model. Our data support the hypothesis that CRH is not only a proinflammatory factor but is also a potent anti-inflammatory factor participating in the regulation of immune system responses to CFA.

More specifically, we first demonstrated that *Crh-/-* deficient mice have lower pain thresholds in response to thermal stimulus following CFA inflammation but not under basal conditions (non-inflamed animals). To our knowledge, this is the first report that has attempted to investigate the antinociceptive effect of endogenous CRH on both non-inflammatory and inflammatory conditions. Previous studies have shown that exogenous CRH can attenuate pain when administered via intraplantar or intraperitoneal injection [23] and that this effect is blocked by the non-specific CRH antagonist ahelical CRF or by blockade of CRFR2 [11]. In our model, lack of both systemic and local CRH resulted in increased pain, which was accompanied by lower corticosterone levels (Table 5). This effect was reversed with the exogenous pharmacological treatment of the mice with glucocorticoid. In all previous studies, the mechanism of analgesic action of CRH involved the release of opioid peptides within the inflamed tissues [24, 25]. In accordance with these published results, we also found that POMC mRNA levels in the inflamed tissue were significantly lower in the *Crh*-deficient mice. However, protein levels of β-endorphin were also lower, but they did not reach statistical significance at any time point tested.

**Table 5:** Corticosterone levels in *Crh* + */* + and *Crh-/-* mice 6 and 24 h following CFA injection

	*Crh* + */* + mice		*Crh-/-* mice	
	6 h	24 h	6 h	24 h
Corticosterone (μg/dl)	10.30 ± 2.64 (*n* = 4)*	7.25 ± 1.14 (*n* = 4)*	2.46 ± 0.56 (*n* = 4)	3.42 ± 0.72 (*n* = 4)

Values are MEAN ± SEM (*n* = No. of mice/group). Data were analyzed using Mann–Whitney. * Depicts statistical significance between genotypes. *P = 0.021

We then investigated the inflammatory response in both genotypes following the induction of inflammation with CFA. To our surprise, both leukocyte infiltration and edema were significantly increased in *Crh-/-* mice compared with their wildtype littermates. We have previously shown that *Crh-*/- mice had a significantly lower inflammatory response compared with the *Crh* + / + mice in the turpentine model of local inflammation [26], a result which was most probably attributable to their CRH deficiency and, thus, lack of peripherally expressed proinflammatory CRH. In contrast, in the current model (CFA-induced local inflammation), the glucocorticoid insufficiency is likely responsible for the observed response. This finding was further confirmed by the fact that glucocorticoid replacement significantly reduced leukocyte infiltration and edema to levels similar to those of *Crh* + / + mice [27].

The CFA-induced inflammation model is accompanied by the production of proinflammatory cytokines, such as IL-6 and TNF-α, [28, 27], which enhance the pain response and sensitize the nociceptors [29]. Furthermore, central administration of IL-6 or TNF-α in rats induces thermal hyperalgesia [30], and injection of TNF-α or IL-6 in the hind paw of rats evokes hyperalgesia, mimicking the actions of carrageenan [31]. Tissue and plasma levels of cytokines (TNFα, IL-1β, and IL-6) from *Crh–/–* inflamed mice were significantly higher compared with those of the *Crh* + */* + mice, in agreement with reports using other models of immune activation [26, 32]. The high levels of the cytokines in *Crh–/–* mice together with their low levels of glucocorticoids suggest that the regulation of cytokines during inflammation is CRH-independent. The above hypothesis is also confirmed by the differences in the cytokine levels of *Crh*-/- mice untreated and treated with glucocorticoid. These findings suggest that production of proinflammatory cytokines is CRH-independent during inflammatory pain unlike in other states of immune system activation [26].

## Conclusions

In conclusion, we demonstrate for the first time, to our knowledge, that *Crh* deficiency is associated with pronociception, which is, however, attributable to some extent to the related glucocorticoid insufficiency. The mechanism of action partially involves the expression of the opioid peptide system in the inflamed tissue, since POMC mRNA expression was significantly lower at the site of inflammation of *Crh-/-* mice. The role of other factors involved in analgesia, such as substance P and NGF, should be should examined in in vivo as well as in vitro studies.

## Data Availability

The datasets used and/or analyzed during the current study are available from the corresponding author on reasonable request.
